# Integrated immunoinformatics and subtractive proteomics approach for multi-epitope vaccine designing to combat *S. pneumoniae* TIGR4

**DOI:** 10.3389/fmolb.2023.1212119

**Published:** 2023-07-25

**Authors:** Sami S. Ashgar, Hani Faidah, Farkad Bantun, Naif A. Jalal, Naeem F. Qusty, Abdulla Darwish, Shafiul Haque, Essam M. Janahi

**Affiliations:** ^1^ Department of Microbiology, Faculty of Medicine, Umm Al-Qura University, Makkah, Saudi Arabia; ^2^ Laboratory Medicine Department, Faculty of Applied Medical Sciences, Umm Al-Qura University, Makkah, Saudi Arabia; ^3^ Department of Pathology, Bahrain Defense Force Hospital, Riffa, Bahrain; ^4^ Research and Scientific Studies Unit, College of Nursing and Allied Health Sciences, Jazan University, Jazan, Saudi Arabia; ^5^ Gilbert and Rose-Marie Chagoury School of Medicine, Lebanese American University, Beirut, Lebanon; ^6^ Centre of Medical and Bio-Allied Health Sciences Research, Ajman University, Ajman, United Arab Emirates; ^7^ Independent Researcher, Jurdab, Bahrain

**Keywords:** immunoinformatics, subtractive proteomics, multi-epitope vaccine, *Streptococcus pneumoniae* TIGR4, molecular docking, molecular dynamics

## Abstract

*Streptococcus pneumoniae* is one of the major precarious pathogens accountable for over 1.2 million fatalities annually. The key drivers for pneumococcal vaccine development involve high morbidity and mortality in over one million cases, especially in very young children and the elderly. In this study, immunoinformatics was integrated with subtractive proteomics to find antigenic proteins for designing a multi-epitope vaccine against *S. pneumoniae*. As prospective vaccine targets, the developed pipeline identified two antigenic proteins, i.e., penicillin-binding protein and ATP synthase subunit. Several immunoinformatics and bioinformatics resources were used to forecast T- and B-cell epitopes from specific proteins. By employing a mixture of five cytotoxic T-cell lymphocytes, six helper T-cell lymphocytes, and seven linear B-cell lymphocyte epitopes, a 392 amino acid-long vaccine was designed. To enhance immune responses, the designed vaccine was coupled with a cholera enterotoxin subunit B adjuvant. The designed vaccine was highly antigenic, non-allergenic, and stable for human usage. The stability of the vaccine with toll-like receptor-4 was evaluated by molecular docking and molecular dynamic simulation. In addition, immunological simulation was performed to test its real-world potency. The vaccine codon was then cloned *in silico*. Overall, this study paves the way for the development of a multi-epitope *S. pneumoniae* vaccine under laboratory conditions. Furthermore, the current findings warrant for the experimental validation of the final multi-epitope vaccine construct to demonstrate its immunological reinforcing capability and clinical applicability.

## Introduction

Pneumococcus, also known as *Streptococcus pneumoniae*, is a Gram-positive bacterium that can cause otitis media (middle ear infection), meningitis, pneumonia, and sepsis. It is a primary cause of illness and mortality globally, especially among small children, the elderly, and people with weakened immune systems (Mahapatra et al., 2021). This Gram-positive bacterium has an extremely high rate of asymptomatic colonization in humans, i.e., up to 95% in babies and 25% in adults (Agnew et al., 2022). With over 100 serotypes of the very diverse bacteria, *S. pneumoniae* can be distinguished from one another by the structure of their polysaccharide capsules (Zahroh et al., 2016; Weiser et al., 2018).

Several virulence factors in *S. pneumoniae* contribute to its pathogenicity. One important factor is its polysaccharide capsule, which assists the bacteria in evading the host’s immune system and increasing its ability to colonize and cause illness. Other factors include pneumolysin (a toxin that destroys host cells), adhesins, and enzymes that allow the bacteria to breakdown host tissues, while evading immune responses (Vollmer et al., 2019; Musher et al., 2022). *S. pneumoniae* is generally spread *via* respiratory droplets, which are typically passed from person to person via coughing, sneezing, or close contact. It generally colonizes the upper respiratory tract asymptomatically, but it might cause infection if it invades other tissues or if the host’s immune system is weakened (Vaughan et al., 2007; Oliver et al., 2017).

The exorbitant expense of antibiotic therapy and increasing antibiotic resistance make the pneumococcus vaccine a particularly alluring strategy. Presently, there are two types of vaccines against *S. pneumoniae* based on a limited number of serotype-specific capsular polysaccharides; i.e., 7-, 10-, or 13-valent pneumococcal conjugate vaccine (PCV), which is made up of the most common polysaccharides conjugated to a carrier protein, and the 23-valent pneumococcal polysaccharide vaccine (PPV), which is made up of 23 different capsular polysaccharides, are in use (Dorosti et al., 2019). The PPV has broad coverage but does not safeguard at-risk individuals under the age of 2 years, whereas the PCV develops effective protective immunity in neonates but has limited coverage, demands high production cost, and needs multiple injections (Silva et al., 2022). Pneumococcal infections have increased due to non-vaccine serotypes that lower the efficiency of this class of vaccines. Hence, in response to previously stated issues, a novel vaccine that is less expensive and offers wide protection against all pneumococcal serotypes is desired. Protein-based pneumococcal vaccines, which comprise a blend of conserved proteins found in a majority or all strains, offer a viable substitute to currently available vaccinations.

Genomic information is widely available to find pathogenic proteins apt for vaccine development. Recently, the combination of immunoinformatics with the subtractive proteomic technique has grown more appealing for the development of effective, affordable vaccines against a variety of infections (Khan et al., 2019). In the present study, subtractive proteomics filters were used to screen potential proteins for vaccine development from the complete proteome. Proteins were then submitted to immunoinformatics tools in order to create an effective *S. pneumoniae* multi-epitope vaccine (MEV). Unlike conventional vaccines, vaccines designed using immunoinformatics are thermodynamically stable and have few or negligible side effects (Gul et al., 2020). Hence, an effective MEV targeting *S. pneumoniae* was designed and validated in terms of its efficacy and stability using a variety of bioinformatics tools and techniques of protein–protein docking, MD simulations, computational expression validation (*in silico* cloning), and vaccine-related immune response simulation. It was found that MEV created in the current work may establish stable connections with human immune receptors and is capable of inducing a successful host immunological response.

## Methodology

### Protein sequence retrieval

The complete proteome sequence in the FASTA format of *S. pneumoniae* serotype 4 (strain ATCC BAA-334/TIGR4) was taken from UniProt. Essential proteins were selected based on their functionality and pathogenicity. UniProt is a comprehensive and freely accessible database of protein sequences and functions. For the prevention of an autoimmune response, the selected proteins must not be homologous with human proteins. Therefore, for finding non-homologous proteins in this research, BlastP was used. BlastP is a popular bioinformatics tool that compares a query protein sequence to a database of protein sequences in order to find similarities and infer functional links. Antigenicity was also assessed through the VaxiJen server (threshold = 0.05), and the protein selected for vaccine development must have a higher antigenicity level. VaxiJen predicts antigenicity using a machine learning algorithm trained on known antigenic and non-antigenic protein sequences.

### CTL epitope prediction

Cytotoxic T lymphocyte cells perform a critical role for the recognition of an antigen. Sequences of the chosen proteins were provided in a FASTA format for the MHC class I epitope prediction using CTLPred, and the consensus approach for epitope prediction was employed. CTL epitopes with consensus scores <2 were chosen for vaccine sequence construction, as the lowest score indicates strong allele-binding ability. CTLPred is intended to discover possible T-cell epitopes within a particular protein sequence that have the potential to bind to MHC-I molecules and be identified by cytotoxic T cells. CTLPred’s prediction algorithm combines sequence- and structure-based features to improve the accuracy.

The VaxiJen 2.0 server was used for antigenicity evaluation, which refers to the ability of an epitope to trigger the immune response. The epitope must be non-allergic and non-toxic in nature, for the prediction of allergenicity and toxicity; AllerTOP 2.0 and ToxinPred servers were used for the same.

### Helper T lymphocyte epitope prediction

Helper T lymphocyte (HTL) cells are the crucial component of an adaptive immune system that are capable of mounting both a cellular and humoral immunological response against foreign pathogens. Consequently, epitopes of HTL coupled with MHC class II alleles are required for the creation of vaccines. T cells aid B cells in secreting antibodies to lyse active parasitic cells in response to CTL cells and macro-phagocyte harmful pathogens. The chosen proteins were subjected to ProPred with a threshold of less than 3. HTL releases several cytokines, including IFN-gamma, IL-4, and IL-10, that activate CTL cells and other cells of the immune system. The epitopes of IFN-gamma are produced by using the support vector machine (SVM) approach, and the IFN epitope server is used to access IFN models. In contrast, for the prediction of inducing properties of IL-4 and IL-10, IL4pred and IL10pred servers were utilized, respectively. Both of these tools are designed for the prediction of peptides that are interleukin-inducing. Utilizing SVM with 0.2 and 0.3 criteria, the IL4pred and IL10pred operations were performed, respectively.

### Prediction and evaluation of B-cell epitopes

B-cells epitopes are necessary for the induction of an adaptive immune response and an essential component of vaccine construct. ABCPred was used for B-cell epitope prediction. ABCPred is a bioinformatics tool that predicts antigenic epitopes or antibody-binding sites inside protein sequences. It focuses on predicting linear B-cell epitopes, which are areas on proteins that are recognized and bound by antibodies. The threshold value was set at 0.5 for predicting B-cell linear epitopes in this tool. Antigenicity, toxicity, and allergenicity were evaluated through the VaxiJen 2.0, ToxinPred, and AllerTOP 2.0 servers of the predicted B-cell epitopes, respectively.

### Sequence construction of MEV

The MEV sequence was constructed by joining adjuvant and epitopes CTL, HTL, and B cell through linkers. An adjuvant that is necessary for modulating the immune response to a vaccine was carefully selected. Thus, the adjuvant used in this vaccine sequence construct was cholera enterotoxin subunit B. At the N-terminal of the vaccine construct, the adjuvant was added through the EAAAK linker. For the effective functioning of each epitope, the linkers’ insertion between the epitopes is essential, which gives an efficient separation among each epitope. All the CTL and HTL selected epitopes were linked together with the help of AAY and GPGPG linkers, respectively. Linear B-cell epitopes were joined together through the KK linker for the conservation of their self-governing immunogenic responses.

### Analyzing the structure

First, the MEV sequence generated was tested for homology against the *Homo sapiens* proteome using BlastP with default parameters. Using the VaxiJen and IEDB servers, antigenicity and immunogenicity profiles were assessed. The modified vaccine’s physicochemical properties, including molecular weight, theoretical Pi, Instability_Index, aliphatic_index (AI), half-life both *in vivo* and *in vitro*, and GRAVY values were ascertained through the ProtParam server. ProtParam is a program that computes various physical and chemical parameters for a particular protein contained in SWISS-PROT or TrEMBL, as well as for a user-entered protein sequence. The vaccine should be non-toxic and devoid of allergic responses. As a result, our vaccine’s toxicity and allergic aspect have been anticipated through the ToxinPred server and AllerTOP server, respectively.

### Prediction of the secondary structure

The MEV construct was determined using PSIPRED for the prediction of a secondary structure, which also assessed the different structural properties of the vaccine like alpha helices, beta turns, coils, and extended chain. This tool uses the ANN machine learning algorithm for predicting the secondary structure of the vaccine construct from its primary sequence.

### Prediction, refinement, and validation of the tertiary structure

The structure of a protein that bends and twists adequately to maintain optimal stability is the 3D structure, which has the lowermost energy value. To enhance protective accuracy of the structure and prediction of the function, our vaccine’s tertiary structure was developed using the Phyre2 server. It uses homology modeling strategies to predict the protein’s 3D structure from its amino acid sequence. The projected 3D structure of our vaccine was improved and enhanced through the GalaxyRefine server. By using the RAMPAGE server outcomes, we validated our refined structure. This was monitored through the ProSA-web server which is a test of structural verification, which yields an actual excellence score utilized to examine information about non-bonded relationships in the structure of the *Mycoplasma* pneumoniae vaccine.

### Prediction of discontinuous B-cell epitopes

The ElliPro tool, which used the tertiary structure of an MEV construct as input and defaulted all the other settings, was used to predict the discontinuous B-cell epitopes of the vaccine design. ElliPro provides the protrusion index (PI) score to each epitope that is being predicted. PI scores of residues are the standards for the identification of discontinuous B-cell epitopes.

### Disulfide engineering of the Refined MEV structure

Disulfide engineering was designed for improving the refined model’s stability before proceeding further. The bonds of disulfide are covalent interactions that stabilize the molecular interactions of the vaccine’s structural model by precising the geometric conformations. The Refined model of a vaccine construct was given as an input in the Disulfide by Design v2.0 server for disulfide engineering. The server predicts disulfide bonds based on the input protein sequence and structural information. It discovers and offers connectivity patterns for pairs of cysteine residues that are likely to generate disulfide bonds.

### Docking between the TLR4 receptor and MEV construct

Once the vaccination protein interacts with host immune cells, a potent immunological response is set up. So molecular docking research was conducted to see how well MEV could attach to human immunological receptors. The toll-like receptor 4 (TLR4) is a member of the family of pattern recognition receptors, which are essential for the process of human resistance and react to extremely responsive and selective foreign microbes. Lipooligosaccharides and lipopolysaccharide are examples of molecular pathogen-associated patterns (PAMPs) that can activate TLR4. After carefully examining TLR4, researchers discovered that it plays a crucial function in triggering an antibacterial immune response. The TLR4 (PDB ID: 4G8A) structure was retrieved and selected as a receptor. To dock MEV with TLR4, the PatchDock server was utilized. PatchDock is a bioinformatics tool for docking protein–protein complexes based on geometric shape complementarity.

### Molecular dynamic simulation

Every *in silico* investigation must include a molecular dynamic component to assess protein–protein complex stability. The stability of protein can be determined by contrasting key dynamics of protein with their usual modes. The common protein mobility inside the interior coordinates was examined using regular mode investigation through the iMODS (NMA) server. The multiplex’s intrinsic motions were measured by the server including eigenvalues, covariance, B-factors, and deformability. Whether a particular molecule will deform at each of the primary chain’s residues determines the primary chain’s deformability. The rigidity of motion is expressed by the normal mode value. If the eigenvalue is small, it is considerably simpler to bend structures, which is directly associated with the energy needed to do so.

### Immune simulation

For the verification of immunological reactions of the developed MEV, an *in silico* technique of immune simulation was accomplished through the C-ImmSim 10.1 server. The online C-ImmSim server allows the user to specify the antigen to be injected as a list of UniProt accession numbers, PDB main identifiers, or a multi-protein FASTA text. The C-ImmSim server was utilized to simulate the body’s immunological reactions after being exposed to the intended vaccine construct. The C-ImmSim server mimics three main modules of the well-designed efficient system of mammals, i.e., the lymph node, bone marrow, and thymus. The following parameters of input were chosen for the immunological simulations: The parameters set as input for the immune simulations were as follows: volume was set to 10, HLA allele A was set to 0101 and 0101, B was set to 0702 and 0702, and DRB 1 was set to (0101 and 0101), step number was set to 100, random seed value was set as 12,345, and injection number was 1, while the other parameters were left as default.

### Reverse translation and optimization of codon analysis

The codon usage in organisms differs from one species to another, while codons that are not adapted may be expressed at a small rate in the host. For enhancing gene expression, the codons would, thus, be improved according to the host’s translational machinery. Reverse translation was performed by using the EMBOSS Backtranseq analysis resource and provides an output as the cDNA sequence which uses a K12 strain of *E. coli* inside a model of prokaryotes, for generating a gene sequence. EMBOSS Backtranseq is a tool in the EMBOSS package, which is a collection of sequence analysis bioinformatics tools. Backtranseq is a reverse translation tool that converts a protein sequence into the equivalent nucleotide sequence. This cDNA sequence was evaluated for the optimization of codons, content of GC, and adaptive index of codon from the GenScript rare codon analysis tool. The ideal score of CAI is 1, and the range of the content of GC is in between 30–70 percent.

## Result

### Protein selection

The complete proteome of *S. pneumoniae* serotype 4 (strain ATCC BAA-334/TIGR4) has 2,109 proteins. The necessary 142 proteins were examined using BlastP for the elimination of human homologs, and 19 non-homologous proteins were found. Based on their antigenicity scores, these 19 proteins were further shortlisted. The top five proteins with high antigenicity values (ranges from 0.90 to 1.50) were chosen (Table 1).

**TABLE 1:** Top five most antigenic proteins and their UniProt accession numbers, names, and antigenicity scores.

S. no.	Accession no.	Protein name	Antigenicity score (VaxiJen)
1	A0A0H2URK1	Pneumococcal serine-rich repeat protein	1.5082
2	A0A0H2URQ5	Cell wall surface anchor family protein	0.9798
3	A0A0H2UQT5	Conserved domain protein	0.9495
4	Q97RU7	Cell division protein DivIB	0.9270
5	P66730	DNA-directed RNA polymerase subunit omega	0.9181

### Appraisal and selection of epitopes of CTL, HTL, and B cell

The selected proteins of *S. pneumoniae* serotype 4 were analyzed for epitope prediction, and 145 CTL epitopes were predicted, while the top five CTL epitopes were chosen according to their high antigenicity, non-allergenicity, and non-toxicity nature (Supplementary Table S1). In the same way, overall 41 distinctive epitopes of HTL were predicted. These HTL epitopes’ capacity to induce cytokines like IL-4, IL-10, and IFN-gamma was evaluated, and six epitopes were ultimately selected for designing the MEV construct (Supplementary Table S2). We have found 50 linear B-cell epitopes, while seven non-allergic, non-toxic, and highly antigenic epitopes were selected for designing the MEV construct (Supplementary Table S3).

### Designing the MEV sequence construct

**FIGURE 1:**
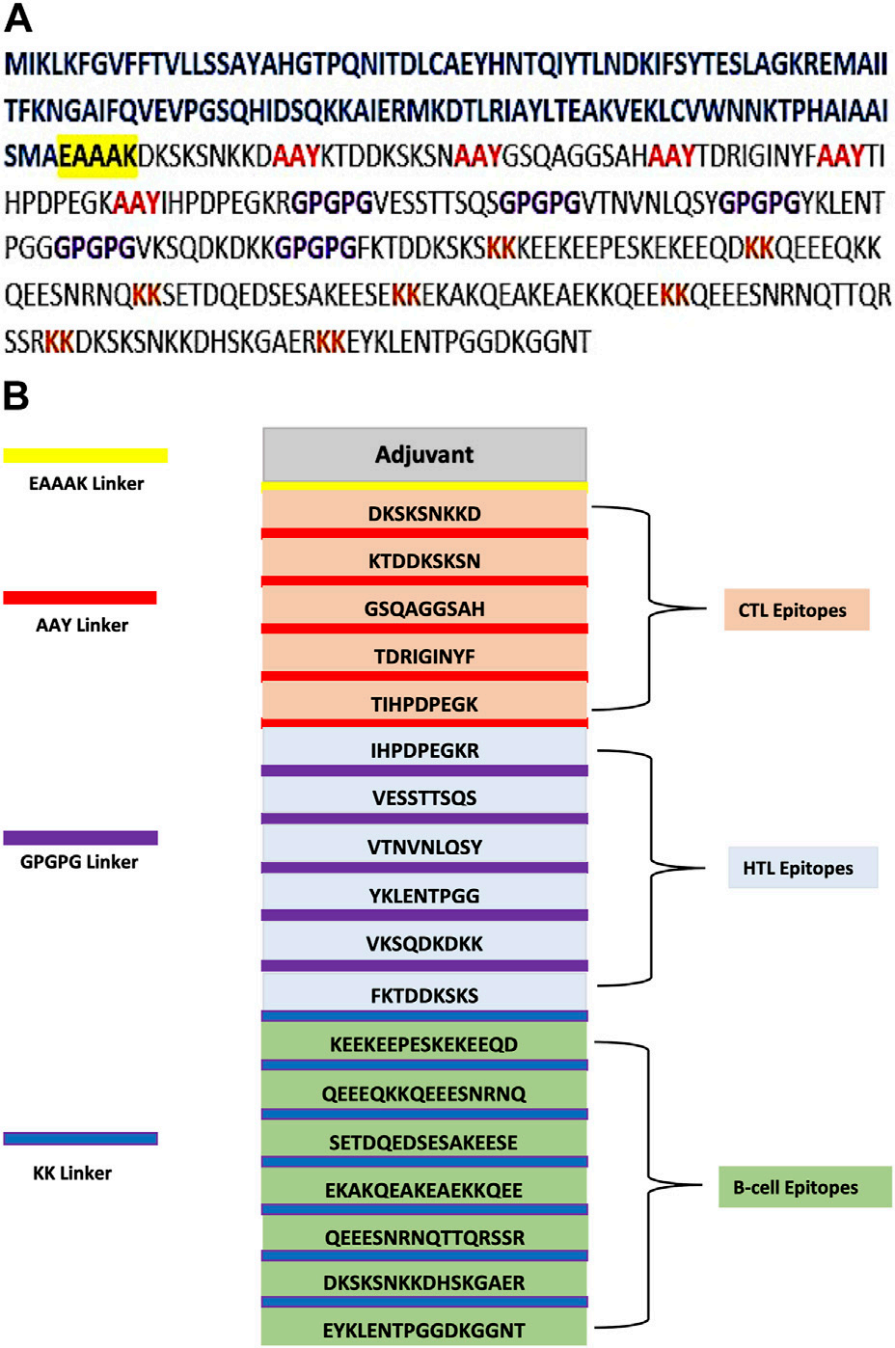
**(A)** Multi-epitope-based sequence construct. Epitope sequence is darkish. Sequence of adjuvant is shown in blue color, the black highlighted in yellow is used for the EAAAK linker, the AAY linkers are in red, the GPGPG connections are in purple, and the KK linkers are in brown. **(B)** Final MEV has 392 residues comprising the EAAAK (yellow) linker that joins adjuvant at the N-terminal. Linker AAY (red) is used for joining epitopes of CTL, and linker GPGPG (purple) is used for joining epitopes of HTL and epitopes of B cell combined through the linker KK (blue).

The selected epitopes of CTL, HTL, and B cell were used to create the final MEV construct; these highly antigenic, non-allergic, and non-toxic epitopes were then connected through their corresponding linkers AAY, GPGPG, and KK, respectively (Figure 1A). Linkers are useful as they support vaccine in epitope presentation and also prevent junctional epitope formation. The linker EAAAK was chosen for joining adjuvant with MEV, as it improves the overall structure’s constancy and reduces protein component joining through efficient detachment. The final vaccine sequence, which is based on many epitopes, has 392 amino acids (Figure 1B) that shows the organization of all the selected epitopes and their corresponding linkers.

### Accessing structural properties

First, the human proteome was used to compare the homology of the created MEV; the outcomes showed that it had no similarity with the proteome of a human. Following this, an examination of the vaccine model’s allergenicity, antigenicity, and toxicity was conducted (Table 2). Our vaccine model is substantially non-allergenic, non-toxic, and highly antigenic. ProtParam explored the physicochemical aspects of vaccine construction. The vaccine’s molecular weight was 43,231.80 KDa, although the theoretical Pi was 8.94 KDa. The half-life of this vaccine design was estimated to be 30 h in mammals (*in vivo*), more than 20 h in yeast (*in vivo*), and more than 10 h in *E. coli* (*in vivo*), and its GRAVY value has been calculated to be −1.341; the vaccine was hydrophilic in nature, as indicated by the value’s negative sign.

**TABLE 2:** Structural analysis of the MEV construct.

Theoretical pI (KDa)	Molecular weight (KDa)	Half-life	GRAVY	Antigenicity	Allergenicity	Toxicity
8.94	43,231.80	Yeast, *in vivo* >20 h *in vitro*	−1.341	1.3730	Non-allergenic	Non-toxic
		30 h, and for *E. coli in vivo* >10 h				

### Analyzing secondary structural properties of MEV

Using an online server called PSIPRED, the MEV’s secondary structure was predicted. Based on their protein’s primary sequence, this tool predicts the secondary structure of MEV. The final MEV’s sequence comprised 165 amino acids that are used to form coils, 198 amino acids that are used to form alpha helices, and 29 amino acids that are used to form beta-strands. The complete prediction result of the secondary structure demonstrates that the value of coils is 42.10%, beta-strands is 7.39%, and alpha helices is 50.51%.

### Prediction of the tertiary structure, its validation, and refinement

**FIGURE 2:**
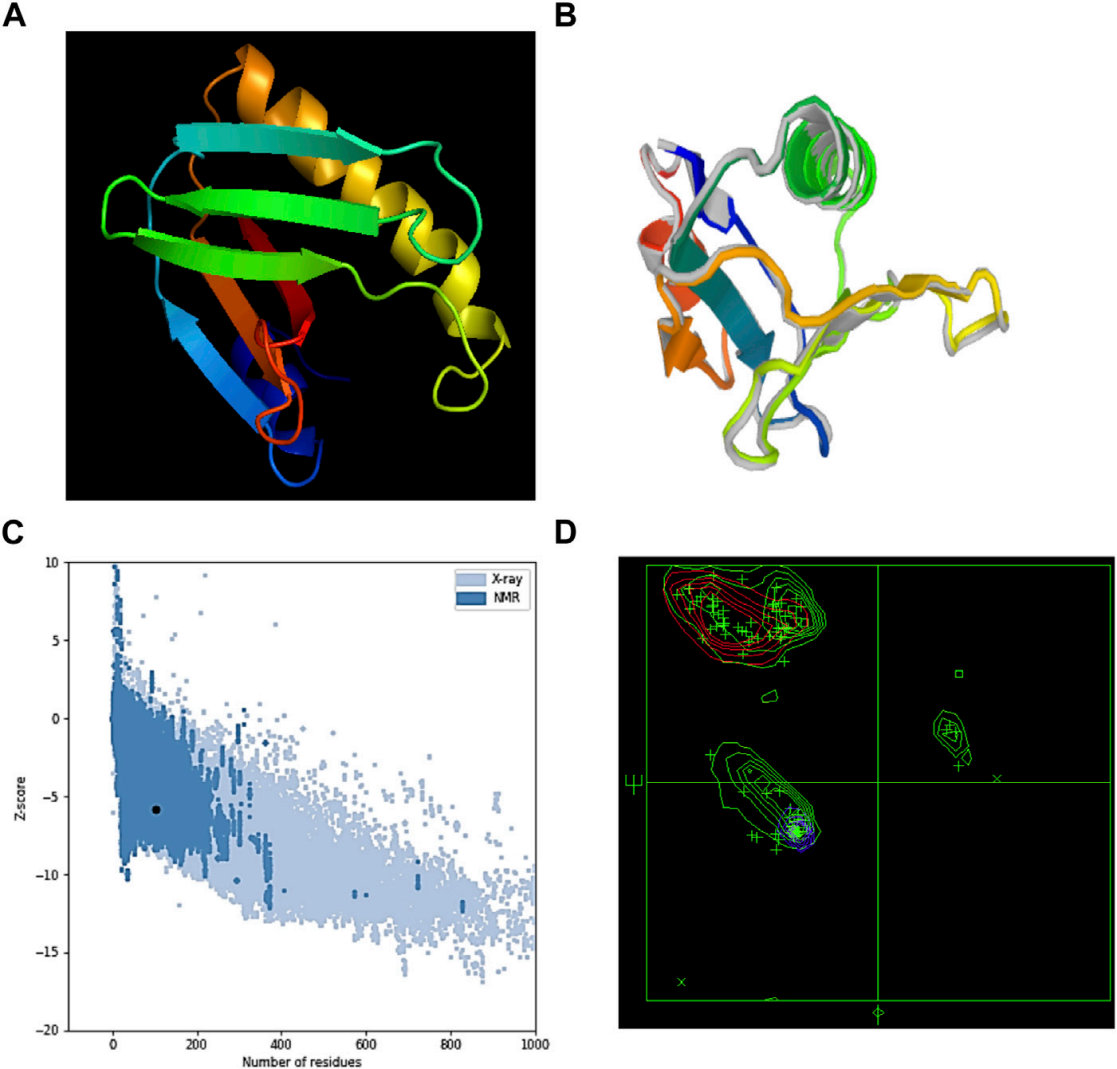
**(A)** MEV’s tertiary structure, **(B)** refined structure of MEV, **(C)** ProSA-web server Z-score, and **(D)** Ramachandran plot result of the MEV’s refined model.

The *S. pneumoniae* vaccination’s tertiary structure was predicted using the Phyre server (Figure 2A). The percentage identity score was 97%. The predicted tertiary structure was optimized through the GalaxyRefine server (Figure 2B). The Ramachandran plot’s upgraded model analysis demonstrated that 99% of residues were located in preferred areas, and 1% were in the permitted areas. Therefore, −5.84 was the resulting Z-score (Figures 2C, D). These outcomes demonstrated the refined model’s high quality.

### Discontinuous B-cell epitopes

**FIGURE 3:**
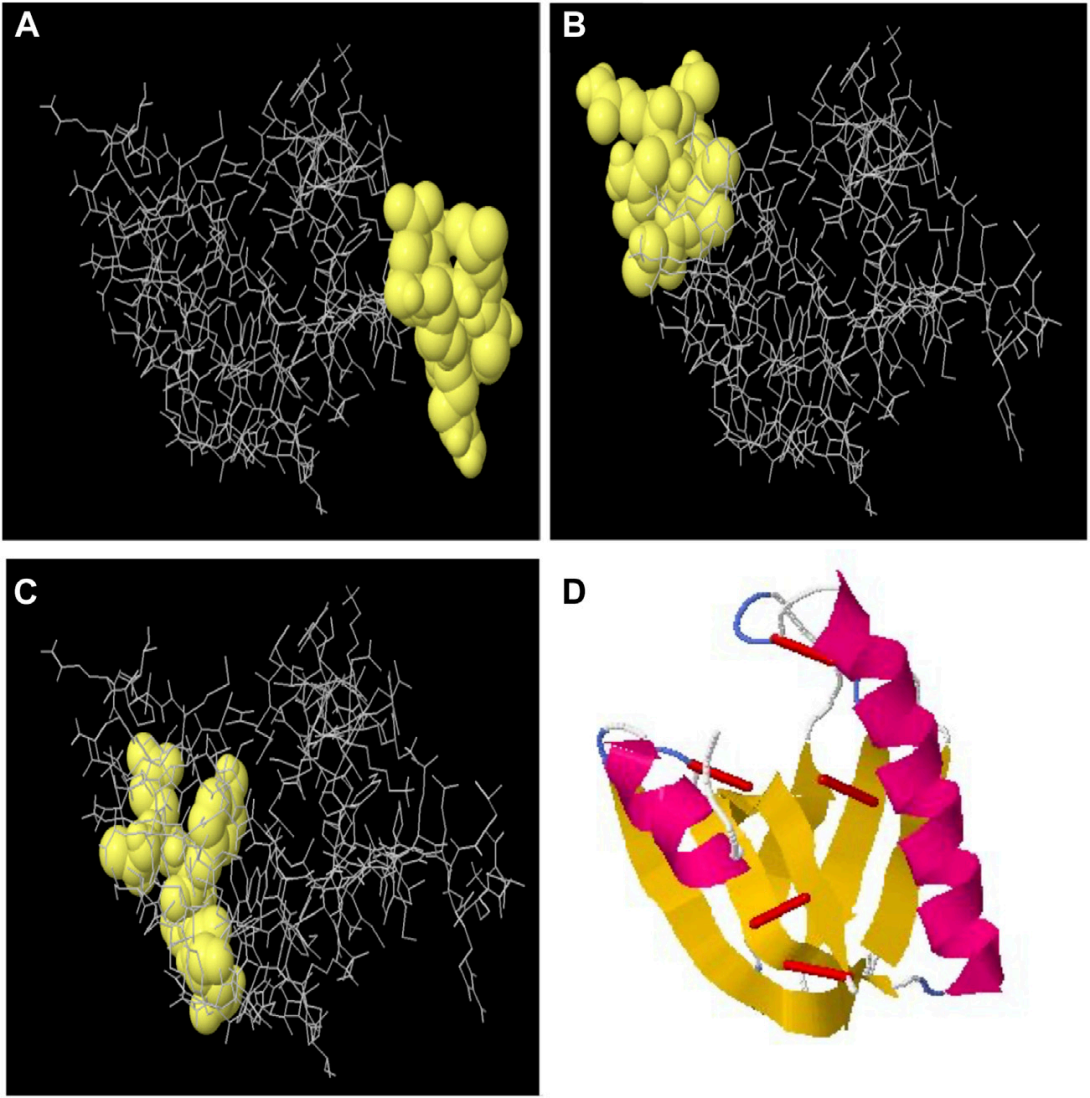
**(A)** Discontinuous B-cell epitope with 0.788 score, **(B)** discontinuous B-cell epitope with 0.766 score, and **(C)** discontinuous B-cell epitope with 0.741 score. **(D)** Disulfide engineering to improve the stability of the multi-epitope vaccine structure. Red colored small rods show the disulfide bonds for five mutated pairs based on energy values and B-factor.

By using the ElliPro server, the discontinuous B-cell epitopes were predicted. The tertiary structure of an MEV was submitted as input for this server and was left as a default parameter. This server predicted the seven epitopes out of which epitopes with a prediction score >0.50 were carefully chosen (Supplementary Table S4) as B-cell discontinuous epitopes (Figures 3A–C). This method identified protruding regions in antigen surfaces.

### Disulfide engineering of MEV

The refined model’s stability was assessed by disulfide engineering through the Disulfide by Design v2.0 server. A total of 34 amino acid pairs were used for disulfide engineering. Seven pairs of amino acids were finalized and selected for disulfide engineering because their energy and chi-squared scores lie between normal ranges. Although, five mutations were formed among the residue pairs which included His78–Gln82 with +115.94 angle and 2.81 kcal/mol of energy, Lys112–Ala116 with −111.50 and 4.05 kcal/mol of energy, GLU104–Ala123 with +114.05 and 5.26 kcal/mol of energy, Ala59–Val71 with +96.34 and 5.24 kcal/mol of energy, and Val108–His115 with +75.60 with 4.20 kcal/mol of energy (Figure 3D).

### Docking between the TLR4 receptor and MEV construct

**FIGURE 4:**
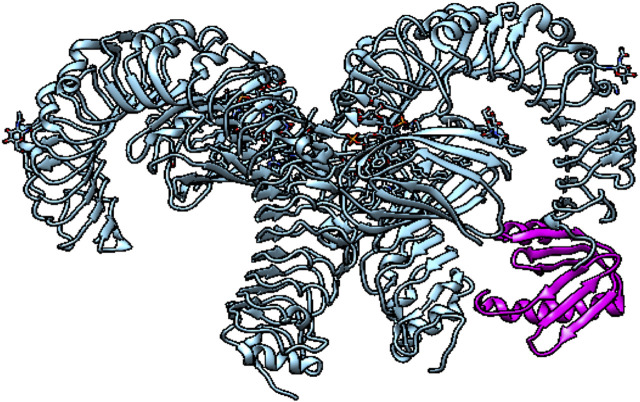
TLR4 with a multi-epitope-based vaccine construct map shown in PyMOL.

The immunological receptor’s molecules and the antigen’s molecules must properly link. Therefore, MEV docking with the human TLR4 immune receptor happens on the PatchDock server. Following bacterial detection, TLR4 is capable of effectively generating an immune response. The docking research revealed a robust interaction between the MEV and TLR4. TLR4 and MEV had a 370.5 ACE value, and the docking score was −282.36. The color of TLR4 is red and MEV is green, as presented in Figure 4.

### Molecular dynamic simulation

**FIGURE 5:**
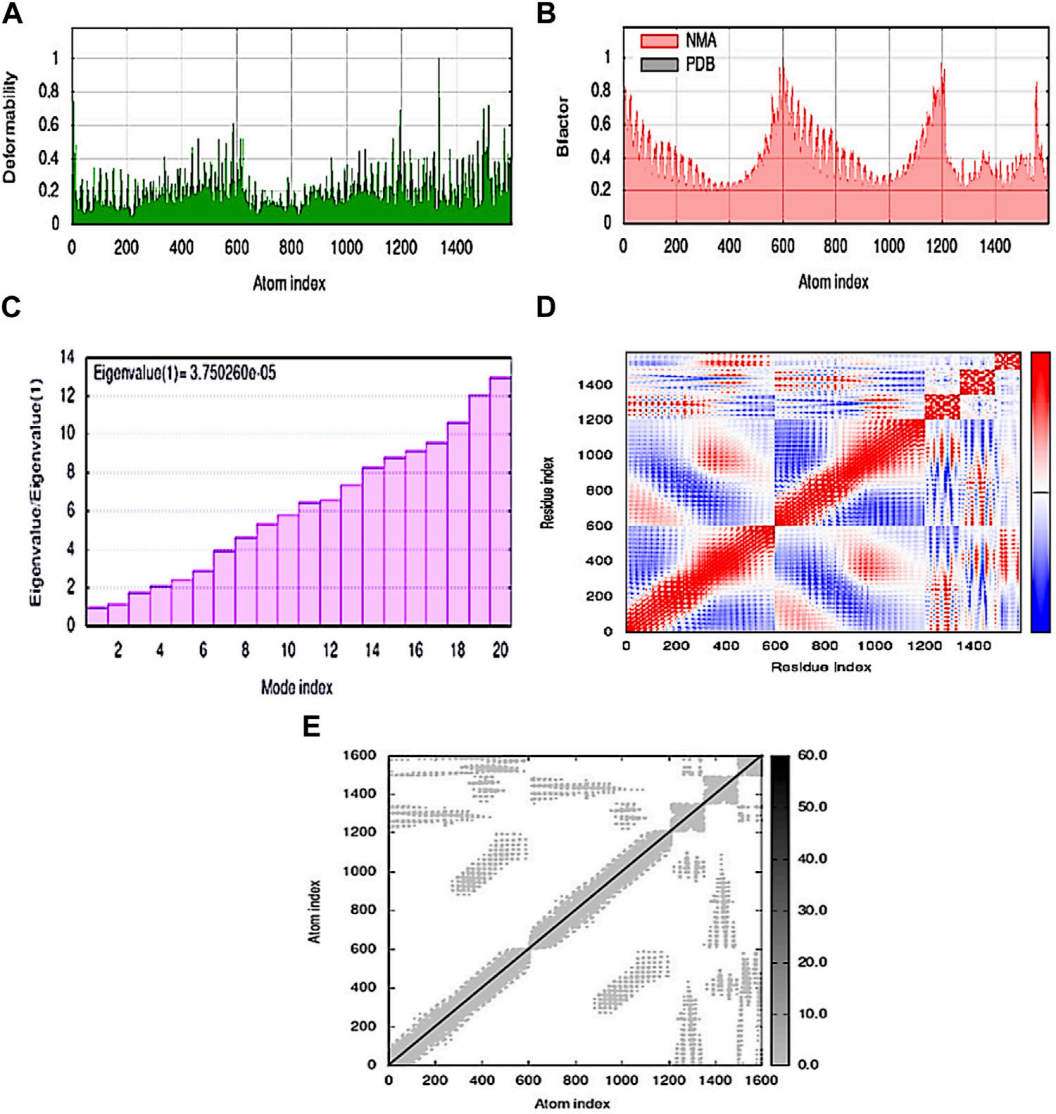
MD simulation of the MEV–TLR4 complex **(A)** deformability; **(B)** B-factor; **(C)** eigenvalue; **(D)** covariance matrix; and **(E)** elastic network map.

Stability and mobility were observed on a broad scale using normal mode analysis (NMA). The IMODS server calculates internal coordinates. Chain hinges validate that the complex’s deformability is dependent on the unique deformation of each residue. The complex’s correct value was 3.750260e-05. Each normal mode’s associated variance was converted to its eigenvalue. The normal mode analysis yielded a B-factor value that was proportionate to the RMS. The covariance matrix shows residue pairs, with unique pairs of related, anti-related, or unrelated motions shown in red, blue, and white, respectively. The Elastic map showed the spring-connected pairs of atoms, with each point designating a spring and gray areas signifying stiffer areas, whose intensity is directly correlated with stiffness (Figures 5A–E).

### Immune simulation

**FIGURE 6:**
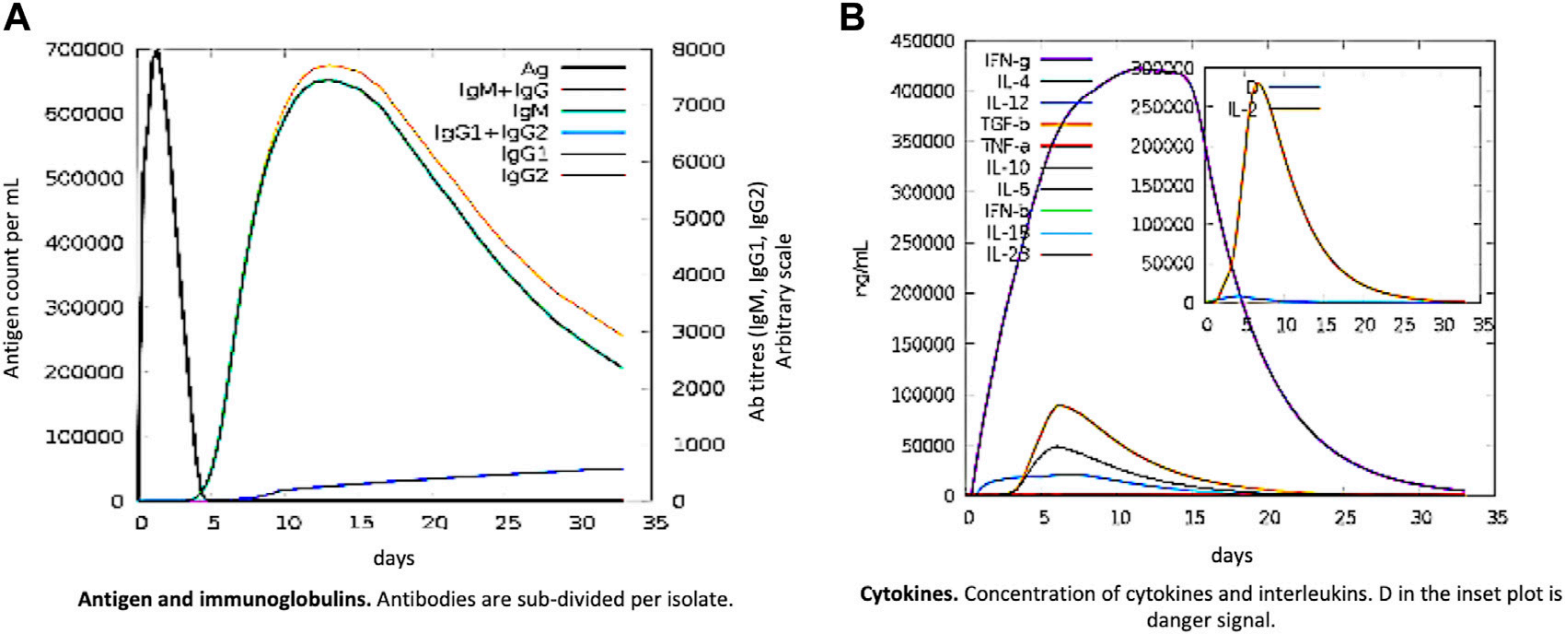
MEV as an antigen: *in silico* immune responses. **(A)** Ig production and B-cell isotypes succeeding the antigen contact in various states using the Simpson Index. **(B)** Cytokine interleukin production.

The pathogen and, probably, the real immune response appear to be significantly impacted by primary and secondary immune responses. The following illustrates the *in silico* host immune system reaction to antigen (Figure 6). Both secondary and tertiary responses showed a normal elevation in immunoglobulin activity, with associated antigen depletion (IgG1 + IgG2, IgM, and IgG + IgM antibodies). Furthermore, there were significant concentrations of cytokines, such as IFN- and IL-2, which are crucial for reducing cellular immunity and viral replication. All these points support an active immune response and subsequent elimination of the MEBV.

### Codon optimization analysis

The preference for particular codons during translation varies among different organisms and is referred to as “codon usage bias.” Codon optimization entails changing a gene’s nucleotide sequence to conform to the host organism’s preferred codon usage. This modification can increase the vaccine construct’s expression levels and translational efficiency in the targeted host system. Codon optimization aims to direct the protein inside the *E. coli* effectively. The *pneumococcus* codons in the MEV construct were modified by the codon used in the K12 strain of *E. coli.* Reverse translation was achieved by using EMBOSS Backtranseq, which provides the cDNA sequence. The improved DNA sequence after codon optimization analysis contains a GC content of 50.18% and a CAI value of 0.96, which was near 1.0, and suggested a satisfactory adjustment (Supplementary Table S5).

## Discussion

Pneumococcal infections are accountable for significant morbidity and mortality, especially in children, and it is expected that an effective vaccination could reduce the death toll (Gharailoo et al., 2016). The existing polysaccharide-based vaccinations are expensive and serotype dependent and their immunogenicity is limited to the serotypes included in the vaccines; however, scientists’ efforts have been concentrated on the creation of serotype-independent vaccines (Norolahi et al., 2020). Pneumococcal protein vaccines are being studied as potential replacements for existing vaccines that can induce serotype-independent protection at a cheaper cost (Lagousi et al., 2019).

The whole *S. pneumoniae* proteome was exposed to subtractive proteomics filters to select the most relevant proteins for vaccine designing. Antigenic proteins are promising candidates for computational vaccine development. Human homologs were identified and deleted to avoid an autoimmune reaction. Five antigenic and non-homologous proteins, pneumococcal serine-rich repeat protein, cell wall surface anchor family protein, conserved domain protein, cell division protein DivIB, and DNA-directed RNA polymerase subunit omega, were picked as a target. The identified proteins were then utilized for predicting the epitopes.

CTL, HTL, and B-cell epitopes were chosen as vaccine candidates via various servers and databases. The MEV construct was created by combining HTL, B-cell, and CTL epitopes with linkers GPGPG, KK, and AAY, respectively. Linkers were employed as an essential component in the production of vaccine proteins to increase expression, stability, and folding. Adjuvant coupling is required for MEVs as they are insufficiently immunogenic when administered alone (Meza et al., 2017). As an adjuvant, cholera toxin B subunit (CTB) was added with the EAAAK linker. CTB has been studied as a traditional mucosal adjuvant that can boost vaccine immunogenicity. It has been used in several studies in the past (Hou et al., 2014). After the addition of linkers and adjuvants, the final MEBSV was discovered to be 392 amino acids long.

Various computational servers were used to evaluate the construct’s physicochemical and immunological properties. The designed vaccine’s molecular weight was 43.80 KDa, making it an acceptable construct because proteins with molecular weights less than 110 kDa are more easily isolated and, hence, considered to be more promising candidates for vaccine development (Sanami et al., 2020). The vaccine’s half-life was determined to be >10, >20, and 30 h in *E. coli*, yeast, and mammalian cells, respectively, indicating the time required for the protein to reach half of its amount after being generated in the cell (Wilkins et al., 1999). Our vaccine model was substantially non-allergenic, non-toxic, and highly antigenic. The vaccine’s GRAVY index was negative (−1.341), indicating that the protein was hydrophilic and had a strong interaction with water molecules, implying good solubility.

A stable link is required between immunological receptors (e.g., TLR4) and the proposed vaccine in order for it to be efficiently delivered inside the host body (Johnson et al., 2010). Molecular docking and MD simulation not only validated the robust connections between the MEV construct and the TLR4 immune receptors but also indicated that very little energy was required for this stable binding. The MEV vaccine can, therefore, effectively bind to immunological receptors, according to these studies. With the introduction of disulfide bridges into the finished MEBSV construct, protein stability was considerably enhanced. This bridging helps in the investigation of the vaccine’s genetic components and considerably improves the protein’s thermostability (Donnarumma et al., 2016).

Ideally, a vaccine must theoretically induce both humoral and cellular immune responses. Our vaccine showed the highest IFN production along with considerable IL-10 and IL-2 activity. Extracellular defense against *S. pneumoniae* is provided by antibodies. Additionally, it has been noted that there are numerous active immunoglobulins (i.e., IgG, IgM, and their isotypes that can be implicated in isotype switching) in the body. Furthermore, irrelevant Simpson Index (D) recommends a variety of immune responses that may be thought of as a subunit vaccine and comprise numerous T- and B-cell epitopes.

Codon optimization of the proposed construct was performed to ensure high-level expression in *E. coli* (strain K12). The total GC content and CAI value of the optimized reverse-translated sequence are related to protein expression (Khan et al., 2022). The multi-epitope protein was expressed at high levels in the bacteria according to the results of the GC content (50.18%) and CAI value (0.96). The results of the immunoinformatics study of the vaccine candidate suggested that this construct would be highly effective against pneumococcus, but additional *in vitro* and *in vivo* laboratory experiments are warranted to corroborate our current findings.

## Conclusion

*S. pneumoniae* infection has formed as a life-threatening disease around the world, infecting a large number of population; millions of cases were stated annually. The present study was designed to create an MEV candidate against *S. pneumoniae*. Immunoinformatics and subtractive proteomics approaches were applied to the proteome of *S. pneumoniae* serotype 4 (strain ATCC BAA-334/TIGR4), and five antigenic proteins were identified as potential vaccine candidates. Three kinds of antigenic epitopes, including CTL-, HTL-, and B-cell epitopes, were discovered from five protein sequences and used to create a vaccine structure. Several physicochemical, immunogenic, and antigenic profiles were computationally evaluated for the designed vaccine. Molecular docking was accomplished between MEV and TLR4 receptors for the purpose of stable interactions between them, and also, the molecular dynamic and immune simulation were performed. Finally, codon optimization analysis was conducted to confirm the translation and expression efficiency of MEV. The current findings conclude that the predicted vaccine construct offers a beneficial effect on *S. pneumoniae* disease handling. However, the effectiveness and safety of MEV must be validated through further laboratory experiments.

## Data Availability

The original contributions presented in the study are included in the article/Supplementary Material; further inquiries can be directed to the corresponding author.
