# Supplementary material for: Integrated immunoinformatics and subtractive proteomics approach for multi-epitope vaccine designing to combat S. pneumoniae TIGR4
Source: Front Mol Biosci. 2023 Jul 25;10:1212119. doi: 10.3389/fmolb.2023.1212119 (PMC10407660; doi:10.3389/fmolb.2023.1212119)
Supplement: Supplementary file 1 [file Table1.pdf]

SUPPLEMENTARY MATERIAL

Integrated immunoinformatics and subtractive proteomics approach for multi-epitope vaccine designing to combat *S. penuomonia* TIGR4

Table S1. The selected CTL epitopes of *S. pneumoniae* serotype 4 antigenic proteins in multi-epitope based vaccine (MEV) construct.

S.No	CTL Epitopes	Percentile Rank	Antigenicity Score	Allergenicity Prediction	Toxicity Prediction
1	DKSKSNKKD	1.00	1.9005	Non-Allergen	Non-Toxin
2	KTDDKSKSN	0.57	2.3978	Non-Allergen	Non-Toxin
3	GSQAGGSAH	0.84	2.5934	Non-Allergen	Non-Toxin
4	TDRIGINYF	0.90	1.3873	Non-Allergen	Non-Toxin
5	TIHPDPEGK	0.73	1.4278	Non-Allergen	Non-Toxin

Table S2. The selected HTL epitopes of *S. pneumoniae* serotype 4 antigenic proteins in multi-epitope based vaccine (MEV) construct.

S.No	HTL Epitopes	Antigenicity Score	Allergenicity Prediction	Toxicity Prediction	IFN Epitopes	IL 4 Prediction	IL 10 Prediction
1	IHPDPEGKR	1.7213	Non-Allergen	Non-Toxic	positive	inducer	Non inducer
2	VESSTTSQS	1.7534	Non-Allergen	Non-Toxic	positive	inducer	Non inducer
3	VTNVNLQSY	1.2664	Non-Allergen	Non-Toxic	negative	inducer	Non inducer
4	YKLENTPGG	1.46	Non-Allergen	Non-Toxic	positive	inducer	Non inducer
5	VKSQDKDKK	1.86	Non-Allergen	Non-Toxic	positive	inducer	Non inducer
6	FKTDDKSKS	1.25	Non-Allergen	Non-Toxic	negative	inducer	Non inducer

Table S3. The selected B-cell epitopes of *S. pneumoniae* serotype 4 antigenic proteins used in multi-epitope based vaccine (MEV) construct.

S.No	B-cell Epitopes	Score	Antigenicity Score	Allergenicity Prediction	Toxicity Prediction
1	KEEKEEPESKEKEEQD	0.82	1.7848	Non-Allergen	Non-toxin
2	QEEEQKKQEEESNRNQ	0.82	2.1786	Non-Allergen	Non-toxin
3	SETDQEDSESAKEESE	0.80	2.0189	Non-Allergen	Non-Toxin
4	EKAKQEAKEAEKKQEE	0.72	1.7942	Non-Allergen	Non-Toxin
5	QEEESNRNQTTQRSSR	0.52	1.9270	Non-Allergen	Non-Toxin
6	DKSKSNKKDHSKGAER	0.72	1.9997	Non-Allergen	Non-Toxin
7	EYKLENTPGGDKGGNT	0.91	2.2296	Non-Allergen	Non-Toxin

Table S4. Conformational B-cell epitopes using ElliPro.

S.No	Peptide	Number of residues	Score
1	ESLAGKRE	8	0.788
2	TPQNITD	7	0.766
3	AEYHN	5	0.741

Table S5. Codon optimization analysis table of final multi-epitope based vaccine construct.

Criteria	Actual Value	Ideal Range	Terms
CAI	0.96	0.8 – 1.0	A CAI of 1.0 is considered ideal. The lower the number the higher the chance that your gene will be expressed poorly.
GC Content	50.18%	30% - 70%	The ideal percentage range of GC content is between 30% and 70%
